# Virtual tourism for older adults living in residential care: A mixed-methods study

**DOI:** 10.1371/journal.pone.0250761

**Published:** 2021-05-20

**Authors:** Alexandra J. Fiocco, Geneva Millett, Danielle D’Amico, Laura Krieger, Yadurshana Sivashankar, Seung Hwan Lee, Richard Lachman

**Affiliations:** 1 Department of Psychology, Ryerson University, Toronto, Ontario, Canada; 2 Ted Rogers School of Retail Management, Ryerson University, Toronto, Ontario, Canada; 3 RTA School of Media, Ryerson University, Toronto, Ontario, Canada; Institute of Mental Health, SINGAPORE

## Abstract

Due to financial and mobility barriers, a majority of older adults living in collective dwellings are no longer able to engage in tourism, a leisure activity that contributes to quality of life and wellbeing. Immersive Virtual Reality (VR) may serve as a programmatic tool to facilitate tourism. This pilot study examined the effects of VR tourism exposure on indices of psychosocial wellbeing among older adults living in residential care. Using a mixed-methods study design, 18 older adults were exposed to VR tourism three times a week, over six weeks. Participants reported decreased anxiety and fatigue immediately following exposure, and increased social engagement and quality of life following six weeks of VR tourism. Qualitative data offered additional insight on the process by which VR tourism may enhance wellbeing. Findings suggest that immersive VR tourism may be a viable program for older adults in residential care.

## Introduction

As population ages, the proportion of individuals living in collective dwellings is expected to rise [1]. Collective dwellings encompass a range of living environments including retirement homes, long-term care facilities, and facilities that offer mixed services [2, 3]. The availability of recreational and leisure programing in collective dwellings plays a crucial role in offering older adults a platform for continued learning and social engagement, which are essential for life satisfaction and wellbeing in late life [4, 5]. In a study of adults aged 55 years and older, it was found that leisure satisfaction accounted for 20% of the variance in well-being, above and beyond satisfaction with standards of living, satisfaction with family relationships, and satisfaction with personal health [6]. It was further reported that the greater the frequency in leisure participation, the higher the leisure satisfaction, suggesting that there is a need to offer older adults the opportunity for leisure satisfaction.

One leisure activity that has not been explored among residents of collective dwellings is virtual tourism. There is a wealth of research suggesting that tourism positively impacts life satisfaction and wellbeing [7]. Tourism enables individuals to escape from the routine of their daily lives, and it may offer opportunities to learn and experience something new, while providing a restorative environment. Tourism has been qualitatively reported by older adults as an activity that contributes to their quality of life [8, 9]. Although the long-term benefits of tourism are unclear, a literature review of 29 studies by Chen and Petrick [7] suggested that tourism may increase positive affect, self-reported health, wellbeing, and quality of life. In a systematic review of 363 research articles, Newman et al. [10] proposed five core psychological mechanisms that leisure tourism may elicit to promote wellbeing: detachment-recovery, autonomy, mastery, meaning, and affiliation. It has also been proposed that older adults may have a unique set of motivations for travel including nostalgia, friendship, learning, escapism, thinking, status enhancement, and physical stimulation [11].

Unfortunately, engagement in traditional forms of tourism may be challenging for older adults to access due to financial limitations, poor health, or disability [12–14]. Such barriers are found to impact decisions surrounding tourism, with increased preference for safe tourism options [13], or decreased participation in tourism altogether [15]. Indeed, in a focus group by the primary author, “inability to afford insurance” and “mobility issues” were found to be the largest barriers leading to decreased tourism among individuals residing in collective dwellings (data not published).

In response to this barrier, immersive virtual reality (VR) may serve as a programmatic tool to facilitate leisure tourism among older adults. VR is the use of digital simulation that enables viewers to interact with an artificial computer-generated virtual environment via an interactive device [16]. The VR experience simulates a real-life environment that facilitates telepresence and mental imagery that is not constrained by physical or geographic boundaries and barriers [17, 18]. While tourist-based organizations have offered information through traditional visual mediums (e.g., websites, 2-dimensional videos), VR differentiates itself by enabling users to embody the tourism content, increasing a sense of presence and perceived interaction with the virtual environment [19, 20].

With the advancement of consumer-ready VR headsets (e.g., Oculus Rift, HTV Vive, Samsung Gear VR, etc.) and sophisticated motion tracking systems, content generators are able to develop novel forms of visual media to produce virtual destinations that provide new capabilities [21]. For instance, innovations in technology have enabled the creation of the “VR Triangle”, intersecting interaction, immersion, and imagination within a virtually-generated environment [22]. In practice, VR applications such as *Jaunt VR* and *Within VR* are publicly available apps that allow users to virtually travel to various destinations around the world without the necessity for physical travel. Through these apps, users can visit museums, heritage sites, and sites that are inaccessible to older adults [23–25]. According to Ulrich [26], the visual delivery of information through immersive VR is able to influence people by evoking emotions through mental imagery. This is accomplished through the delivery of heightened visual, auditory, and contextual cues [27]. Accordingly, developers have the capacity to create virtual environments that allow users to experience tourism in an immersive 360° environment, which may enhance the viewer’s comprehension and memory of the visual content, while stimulating an emotional response, all of which may provide a unique experience for older adults in collective dwellings.

To date, only one other study has examined momentary changes in affect in response to immersive VR exposure. In a recent observational study by Brimelow et al. [28], 13 older adults from a 56-bed residential aged care facility were exposed to a 4- to 5-minute immersive VR environment of a relaxing scene. Apathy and emotion in response to immersive VR were indexed using observational measurement tools including the Person-Environment Apathy Rating Scale (PEARS) and the Observed Emotion Rating Scale (EORS). Although researchers failed to observe a significant change in emotions of pleasure, anger, anxiety, sadness, and general alertness, as indexed using the EORS; researchers reported a significant improvement in apathy, evidenced by observed changes in participants’ facial expressions, eye contact, physical engagement, verbal tone, and verbal expressions [28]. Additional research is needed to examine the utility of immersive VR among older adults in collective dwellings, to assess potential risk for cybersickness, and to capture the lived experience of immersive VR from the perspective of the older adult.

The objective of this pilot study was to examine the immediate and longer-term benefits associated with VR tourism among older adults living in residential care. Immediate benefits included perceptions of enhanced happiness, engagement, and relaxation following the immersive VR session. Furthermore, symptoms of cybersickness were investigated given the paucity of research on cybersickness in response to immersive VR within older adult populations. Longer-term benefits were indexed by self-reported changes in social engagement, quality of life and psychological wellbeing following six weeks of VR tourism. It was hypothesized that immersive VR tourism would enhance momentary affect and would not result in significant cybersickness. It was also hypothesized that 6-week exposure to a VR tourism program, which entailed VR tourism exposure three times a week, would increase perceived quality of life, social engagement, and psychological wellbeing. To gain insight on quantitative findings, qualitative interviews were conducted to capture the lived experience of VR tourism among older adults living in residential care. Further, qualitive data were collected to help define existing challenges and areas of improvement for the implementation of VR tourism in collective dwellings.

## Materials and method

### Participants

A total of 18 residential care homes in the Greater Toronto Area (GTA) were emailed an invitation to collaborate on the study. Of the 18 homes that were emailed, six responded and agreed to collaborate. Once the residential care homes were identified, the research team entered each home and gave a general presentation of the study, offering residents an opportunity to try the VR headset. Residents who showed interest in the study were provided with the consent form to review and were told to contact the research team if they wanted to participate in the study.

Twenty-five participants were recruited from the six residential care homes. Exclusion criteria included the inability to provide informed consent, and the presence of immersive VR contraindications including self-reported frequent migraines and self-reported proneness to motion sickness. Participants provided written consent to complete three stages of the study protocol: a Pre-VR tourism assessment, participation in the 6-week VR tourism program, and a Post-VR tourism assessment.

Seven participants withdrew from the study and therefore their data was removed as indicated in the consent procedure (28% attrition). The primary reason for withdrawing from the study was due to medical reasons unrelated to the study; however, one participant withdrew because they found the VR headset uncomfortable, one participant withdrew due to disappointment with the virtual content, and one participant was forced to withdraw as it was later discovered that they met exclusion criteria (i.e., suffered from frequent migraines). The final sample consisted of 18 participants (Mean age = 83.6, SD = 6.17; 38.9% female, see Table 1).

**Table 1:** Participant demographic information (N = 18).

	Mean (SD) or %
Age	83.6 (6.17)
Sex (% female)	38.9
Years of education	19.22 (8.48)
Highest education level attained (%)	
Elementary or less	0
High school	11.1
Some college or technical school	27.8
University	11.1
Graduate	50
MMSE	27.750 (1.7)
Length in current residential setting (months)	28.06 (20.52)
Length in any residential setting (months)	31.72 (26.04)
Self-reported health (%)	
Very poor	11.1
Fair	22.2
Good	33.3
Very good	27.8
Excellent	5.6
Number of Exposure Sessions	17.28 (1.02)

*SD* = Standard deviation; MMSE = Mini-Mental Status Examination.

### Procedure

The current study was approved by the Ryerson University Research Ethics Board (REB# 2016–218). Pre-VR tourism assessment and Post-VR tourism assessment took place in a quiet room at the residential care home and was conducted by a trained graduate student. During the Pre-VR tourism assessment, participants completed the provision of consent, completed a demographics questionnaire, and completed a psychosocial questionnaire battery. Capacity to provide consent was determined by score on the Mini-Mental Status Exam (score > 24/30). During the Post-VR tourism assessment, participants completed the psychosocial questionnaire battery a second time and engaged in a semi-structured interview, to discuss the participants’ experience with the program.

The 6-week VR tourism program involved exposure to immersive VR tourism sessions, three times per week over six weeks. Each VR exposure was six to 10 minutes in length. On each of the scheduled days, participants were asked to visit the designated lounge area of their residential care facility during a specific time of day (a one-hour time slot was created for each of the exposure days, between breakfast and lunch). Participants engaged with the VR content on a first come-first serve basis. A total of three VR headsets were available during each session. Two trained research assistants facilitated each session by collecting momentary data (pre-post VR exposure), cleaning equipment following individual use, setting up VR content for each participant and troubleshooting any technical difficulties. Upon arrival to the lounge, participants were asked to take a seat and to complete questions pertaining to momentary affect and cybersickness. Participants were then provided with the menu of VR travel destinations (and a reminder of destinations previously visited) and fitted with the VR headset and earphones. Following VR exposure, participants were asked to complete the same questions pertaining to momentary affect and cybersickness.

### Measures

#### Quality of life

Quality of life was measured at pre-VR tourism program assessment and at post-VR tourism program assessment using the Older People’s Quality of Life Questionnaire (OPQOL-35; [29]). The OPQOL-35 is a 35-item self-report questionnaire that assesses participants’ levels of satisfaction with their quality of life in various domains including life overall, health, social relationships, independence and control, home and neighbourhood, psychological and emotional well-being, finances, leisure, and religion. Responses were made on a 5-point scale ranging from “strongly agree” to “strongly disagree.” Possible scores range from 35 to 175, with higher scores indicative of greater quality of life.

#### Social engagement

Social Engagement was measured at pre-VR tourism program assessment and at post-VR tourism program assessment using the Social Engagement Scale (SES). The SES is a 5-item scale adapted from Krueger et al. [30], which measures frequency of engagement in activities outside of the residence with family or friends, engagement in church or religious activities, engagement in sports or physical activity, and engagement in other recreational activities. Participants are required to indicate the frequency with which they have engaged in each of the aforementioned activities in the past two weeks on a 4-point scale with the following four response options: zero times, one to two times, three times, or four to five times. Possible scores range from 0 to 13, with higher scores indicated greater social engagement.

#### Depressive symptoms

Depressive symptoms were measured at pre-VR tourism program assessment and at post-VR tourism program assessment using the Geriatric Depression Scale (GDS). The GDS is a 15-item measure of depression in older adults [31]. Participants are asked to respond “yes” or “no” to questions that tap into mood. Possible scores range from 0 to 15, with higher scores indicate of greater depressive symptoms.

#### Momentary affect and cybersickness

Momentary affect and cybersickness was measured before and following each VR exposure session during the 6-week program. Assessment of momentary affect in response to VR exposure was measured using a Visual Analogue Scale (VAS) consisting of three dimensions of affect: unhappy/happy, bored/engaged, and anxious/relaxed. Cybersickness in response to immersive VR exposure was assessed using a modified version of the Simulator Sickness Questionnaire (SSQ; [32]). Six items of the original 16-item questionnaire were chosen (general discomfort, nausea, fatigue, headache, eye strain, and dizziness) to minimize burden of reporting over the 6-week program. Greater score on the SSQ is indicative of greater cybersickness.

#### Qualitative interviews

Semi-structured one-on-one interviews were audio recorded and transcribed verbatim. Interviews consisted of open-ended questions that elicited discussion regarding individual experiences of the VR tourism program and thoughts on VR technology.

### Immersive VR apparatus and tourism content

VR content was administered using the Samsung Gear VR headset, with Samsung Galaxy Note 7 mobile phones and Sony headphones. A helmet-mounted display with headphones helps users feel a sense of isolation from their physical environment, creating a greater sense of immersion and presence. Eighteen 360-degree videos, six to 10 minutes in length, were offered. Travel destinations included: Amsterdam, Australia, Cancun, Canyon Walk, Elephants and Orangutans, Egypt, Far East, Greece, Hong Kong, the International Space Station, Ireland, Paris, Portugal, Prague, Morocco, Scuba Diving, Spain, and Turkey. The videos were curated and chosen to represent a range of tourism activities (e.g. from visiting a city to scuba diving) that allowed for changing view points in response to the viewer’s head movements and did not contain sudden scene changes which could elicit cybersickness.

### Data analyses

To assess change in each outcome measure following the 6-week VR tourism program, 2-tailed paired t-tests were conducted. One exception was the OPQOL-35, which violated assumptions of normality, and thus the Sign Test was used. To assess change in momentary affect and symptoms of cybersickness, an average score over 18 sessions was created for each VAS scale score and for the modified SSQ score at pre- and post-VR exposure. To assess for change in momentary affect and cybersickness in response to VR exposure, 2-tailed paired t-tests were conducted for each of the averaged scores. A *p*-value < 0.05 was chosen to detect statistical significance for all analyses, using SPSS Statistics v23.

Thematic analysis was performed by two independent coders who extracted codes, themes, and subthemes from 18 transcribed interviews. Coders first reviewed the transcripts to become familiar with the content; extracted codes; and generated themes. Themes were reviewed during a recursive process with the coded data and salient extracts were selected.

## Results

### Participant characteristics

Detailed in Table 1, 94.4% of participants described themselves as previous “frequent travellers”. A majority (94.4%) disclosed that they no longer travelled for various reasons including financial and insurance restrictions, health, companion-related concerns, and mobility issues. Participants completed an average of 17.28 (SD = 1.02) VR exposure sessions.

### Momentary affect and cybersickness

Paired t-test using the average SSQ score over 18 sessions revealed a marginal decrease in cybersickness from pre- to post-VR exposure (*t*(17) = 2.07, 95% CI [-0.002, 0.22], *p* = 0.05). This pattern was driven by the SSQ fatigue symptom, *t*(17) = 4.31, 95% CI [0.07, 0.20], *p* < 0.001), suggesting a decrease in feelings of fatigue following VR exposure. Paired t-test also revealed a significant decrease in anxiousness (i.e. increased relaxation) pre- to post-VR exposure, *t*(17) = 3.20, 95% CI [2.40, 11.70], *p* = 0.005. No statistically significant changes were found for unhappy/happy or bored/engaged. See Table 2.

**Table 2:** Average cybersickness and momentary affect scores pre- and post-VR exposure (*N* = 18).

Variable	Pre-VR		Post-VR			
	*M*	*SD*	*M*	*SD*	Change	*p*^a^
Total SSQ	0.51	0.68	0.40	0.59	-0.11	0.05
SSQ Subscale						
Discomfort	0.11	0.19	0.07	0.13	-0.04	0.08
Nausea	0.01	0.02	0.03	0.06	0.02	0.18
Fatigue	0.27	0.41	0.14	0.31	-0.13	**<0.001**
Headache	0.01	0.03	0.03	0.08	0.02	0.54
Eyestrain	0.05	0.10	0.07	0.13	0.02	0.43
Dizziness	0.07	0.22	0.10	0.21	0.03	0.36
VAS Subscale						
Happy	69.23	15.87	74.47	16.20	5.24	0.08
Excited	61.14	18.97	64.63	16.30	3.49	0.12
Anxious	30.68	17.60	23.63	15.17	-7.05	**0.005**

*M* = Mean; *SD* = Standard deviation; SSQ = Simulator Sickness Questionnaire; VAS = Visual Analogue Scale.

^a^ Significance values derived from paired *t*-tests. Bolded *p*-values are significant at *p* < 0.05.

### VR tourism programming for psychosocial wellbeing

Paired t-tests revealed a significant increase in quality of life (*t*(17) = -7.55, 95% CI [-17.70, -9.97], *p* < 0.001) and social engagement (*t*(17) = -2.65, 95% CI [-2.20, -0.25], *p* = 0.02). Paired t-tests failed to show a significant change from pre-VR tourism program assessment to post-VR tourism program assessment in depressive symptoms (*t*(17) = 1.06, 95% CI [-0.55, 1.66], *p* = 0.31). See Table 3.

**Table 3:** Change scores in quality of life, social engagement, and depressive symptoms following the 6-week VR tourism program (*N* = 18).

Variable	Pre-VR program		Post-VR program			
	*M*	*SD*	*M*	*SD*	Change	*p*^b^
OPQOL-35	107.83	10.08	121.67	12.83	13.84	**<0.001**
SES	4.00	2.33	5.22	2.65	1.2	**0.02**
GDS	3.17	2.68	2.61	2.75	-0.56	0.31

*M* = Mean; *SD* = Standard deviation; OPQOL-35 = Older People’s Quality of Life Questionnaire; SES = Social Engagement Scale; GDS = Geriatric Depression Scale. Change score was calculated by subtracting pre-VR tourism program score from post-VR tourism program score (i.e., T2 –T1).

^**a**^ Significance for home subscale of OPQOL-35 derived from Sign test.

^**b**^ Significance values derived from paired *t*-tests.

Bolded *p*-values are significant at *p* < 0.05.

### Qualitative findings

Qualitative interview data from 18 semi-structured interviews fell into two main themes: participants’ personal experience with tourism and virtual tourism, and feedback regarding the implementation and content of VR tourism videos.

#### Personal experience with tourism and virtual tourism

Three sub-themes were identified within the theme of personal experience with tourism and virtual tourism: 1) experience with tourism, 2) short term benefits of virtual tourism, 3) and the opportunity to learn and reminisce through virtual tourism.

*Experience with tourism*. Participants had varying levels of travel experience prior to the study, with some participants reporting frequent tourism around the world, and others noting that they had never travelled outside of their home town. Regardless of the level of past tourism, all residents reported that their current travel was minimal and mostly consisted of visiting family within southern Ontario, or outings with their retirement home to the local mall. All participants voiced that tourism was an activity that they would like to engage in, but were unable to do so due to financial or mobility constraints. One participant shared “I did do travelling… although my daughter, she thinks we should go to [foreign country] … she’d have to put me in a wheelchair, I don’t know. Anyways I consider my travelling days are over” (female resident, 84 years old). Another participant shared “I have multiple sclerosis and … for an unbelievable immediate time space, I have become so much more disabled. In terms of being physically able to be mobile, I can’t. Like yesterday they went to [name of park in the city] and I didn’t go because they couldn’t find out whether they had a disabled bathroom!” (female resident, 88 years old). Overall, participants’ thematic accounts presented challenges with the ability to travel, even to engage in local tourism. Due to these challenges, participants expressed that virtual tourism addressed a need within residential care settings. Regardless of past travel experience, current tourism was seen as an activity that participants enjoyed and would like to engage in. One participant shared “I stopped travelling in my 30s, for various reasons, and, um, so it was nice to see the world again” (male resident, 77 years old).

*Benefits of virtual travel*. Participants reported that virtual travel was something for them to look forward to, that it promoted positive emotions, and that they were immersed in the VR tourism content. A number of participants reported that they looked forward to participating in each VR tourism session. One participant stated “Well, that 10 minutes was pretty… exceptional! [..] I looked forward to it, before each one” (female resident, 71 years old). Another participant shared that the program was interesting and different from their usual scheduled programming, stating “it [VR tourism program] was interesting to do because I don’t go to any of their [resident programming] stuff… they have bingo and they have people coming in to entertain and all that. I don’t do any of those” (female resident, 80 years old); along a similar vein, another participant shared “You know, you go into different activities, and this is the one I was looking forward to for 6 weeks!” (female resident, 86 years old).

An important element of the program which served to promote anticipation was the ability to see something new and exciting each week, and that it broke up the monotonized and predictable schedule of their current setting. One participant shared “Well, I put in most of my time sitting in a chair watching television, so it gave me something to look forward to and to know that I was going to be able to do something else today […] the anticipation is what was wonderful for me. The anticipation that I was going to be able to look at something, and I wouldn’t know what it was until I got here” (female resident, 90 years old).

Residents also reported that they enjoyed virtual travel and that they felt that the activity lifted their spirits. Namely, participants reported that they “really loved” participating in virtual tourism, and that it had “uplifting value” such that they felt happy leading up to, during, and after participating. One participant shared “I came out of there [VR tourism viewing] and I was like, on a high! […] I was happy” (female resident, 86 years old), and another participant expressed “I found most of the tapes energized me and that energy level carried through to the rest of the day” (male resident, 77 years old).

The level of immersion experienced by participants was an important contribution to their level of enjoyment. Residents reported that they felt transported to other places and could almost feel the physical sensations of being there. One participant shared “I felt privileged as if I was exported to other places…the videos were very realistic…I could feel the sand in my toes” (female resident, 90 years old). Another participant expressed “I really got into it. I really loved it. I did feel that I was really travelling because I lost complete sense of where I was. If my hand hadn’t automatically touched the chair [laughs]… like it stunned me for a minute” (female resident, 86 years old). Additionally, greater levels of immersion increased the ability of VR to serve as a distraction and contributed to a sense of “escaping” from the routine of residential care living. One resident reported “I’m handicapped because I can’t walk, ya know. Three times a week I was able to escape and travel to some parts of the world […] when I would be watching, I would notice that my pain threshold was minimum. I didn’t feel as much aware of my pain as I was watching, I escaped” (female resident, 90 years old).

Although participants shared several immediate benefits from engaging in virtual tourism, long-term benefits of VR tourism were less clear. One participant stated “I only see virtual reality as a tool. It’s a help, that’s all. It does not cure depression, or anxiety…it broadens my vision” (male resident, 83 years old). However, another participant shared “I think it contributed to doing other things that made me feel good. So I think, in terms of its general effects for people right now, it could have quite a range. Some people might find it very encouraging, and exciting” (female resident, 81 years old). One benefit that a majority of participants reported was that the VR tourism program provided a conversation piece to discuss with family and friends, and a topic to write about for residents who were part of a writing group. One participant shared that she was now able to virtually visit places that her husband had travelled to in the past; she shared “it may have been obvious to my husband that I was very upbeat when I knew I’m going into a session. He could see I was excited. He travelled the world before he met me. Every time I mentioned it, [husband would say] I’ve been there. Well good. Well now I’m going!” (female resident, 86 years old).

*Discovery and Reminiscence*. Virtual travel presented something different for experienced travellers and for those who had not travelled in the past. For the inexperienced traveller, the VR tourism program provided an opportunity to learn, see, and experience something new. Participants reported the enjoyment of being able to virtually travel to destinations that they had never seen, and would never have an opportunity to see otherwise. In discussing potential advantages of virtual tourism, one participant noted “Visiting countries that I’ll never, never visit… the space station, things like that that I’ll never see” (male resident, 86 years old). When virtually touring new places, participants reported the experience as a learning activity, such that they were able to learn about a place that they had never visited before. One participant noted that they had “learned a lot, especially the different places… I was really absorbed in a lot of it” (female resident, 84 years old), and another participant shared “Just being there [the Great Barrier Reef], even if you’re not a snorkeler or diver… things that you would never do in your real life, they’re approachable by this, that’s what I really enjoyed about that” (male resident, 83 years old). Participants also reported enjoying seeing different ways of living and architecture, and that it broadened their vision of the world. For example, one participant shared “To me it was great seeing parts of the world I had never been to. They opened me up to seeing people living in a totally different lifestyle […] the buildings were great to see too, but to see the people and markets… something totally different from here” (male resident, 96 years old).

For those who had previously travelled to the selected destinations, participants reported that it brought back memories, provided comfort, and that it added another dimension to their tourism experience. One participant shared “I have travelled quite a lot, been all across the world…so it was interesting in that respect, you know? Because some of the things, I knew about it, but it also added another dimension” (female resident, 71 years old). Further, some participants were able to see sites that they had not seen during previous travels, whereas others felt as though they were revisiting; as one participant shared “In the VR I felt like I had visited back” (male resident, 79 years old). Two residents reported that they were “sad” that they could not travel to these places anymore, however they still appreciated the ability to travel to them virtually.

#### Feedback on virtual tourism

After participating in six weeks of virtual travel three times a week, participants had several points of feedback regarding VR for tourism, including strength of the video content and areas for improvement. Residents also voiced their anticipation for VR in the future in the hopes that it can further address the travel needs for older adults in residential care settings.

*Strengths of video content*. Frequently reported favourite virtual travel destinations included *Elephants and Orangutans*, the *Great Barrier Reef*, and *Prague and Spain*. These locations were referenced due to their creative filming, novelty, scope, and their level of immersion. One participant shared “[…] my favourite one would be the elephants and orangutans. I loved it so much and that elephant, when it started walking towards you…and it was so beautifully done and it was like he was going to step right on your head!” (female resident, 71 years old). Others specified that they enjoyed seeing people engaging in culturally relevant activities, “real people doing real things” (male resident, 86 years old), so that they could get a sense of different ways of life. Another participant noted that having a narrator was essential. Specially, they shared “I think Paris was good because they actually had an interviewer who was giving you background” (female resident, 80 years old). Finally, some participants shared that having good quality music that complimented the destination helped to bring the VR experience alive; one participant shared “I found the music would lift it up so much, that it would be fun to watch” (female resident, 71 years old).

*Areas for improvement*. Suggestions for improvement revolved around the need to increase contextual cues and information in the videos, the importance of culturally appropriate music, quality of the images and length of videos to capture the scope of each destination.

Participants reported that having informative labels, descriptions or auditory commentators is necessary to promote learning, enjoyment and immersion. One participant shared “I needed to know exactly what I was looking at, for instance, in many of the places, they did not identify what it was we were looking at… some of the buildings that we saw, there were a lot of buildings that were not identified” (female resident, 88 years old).

As much as music could enhance the virtual experience, it could also compromise the experience for some participants. Participants expressed that the music was very important to their overall experience; however, in several videos, the music was not relevant to the virtual destination (e.g., Johnny Cash in Hong Kong). Some participants reported that music is very specific to each culture and having culturally appropriate music playing during each destination is important to the experience. In fact, participants voiced that incongruent music brought them out of the experience, and when the music was congruent, the quality of the content was less vital. In sharing their experience of the Hong Kong video, one participant expressed “I thought I would know almost right away by listening to the music whether I was going to enjoy it or not… the first one for example was Hong Kong […] the film disappointed me because all you could see in my mind was the tops of buildings and for me […] I thought the music was inappropriate… I said “my God, this is rock and roll.” And it just didn’t suit the film at all … it should be very beautiful Chinese music” (female resident, 71 years old).

A final area of improvement was the scope of content within each video. Especially for shorter videos, participants reported that they did not feel that they were able to experience much of the destination. Participants wanted a more wholistic perspective of each location. One participant noted “I think the length of the video was not long enough. Yeah, so it should have been much longer because, Australia being so big, there are more things to look at than the opera house” (male resident, 75 years old). Another participant noted “They were all interesting. I just find that […] lots of times it didn’t show what I was expecting of a place” (female resident, 86 years old). Overall, participants reported that they would appreciate longer videos as some were less than ten minutes, and that the VR quality could be improved with respect to clarity of the images.

*Hopes for virtual reality in residential care*. Participants understood the preliminary nature of VR tourism in residential care settings. They were provided the opportunity to share hopes for virtual tourism in the future. Participants also suggested the possibly of recording live events in VR so that they can be appreciated by those who cannot attend these events in “real time”. In discussing the ability to see a local opera, one participant noted “I wouldn’t mind seeing a metro opera live in New York that I could see here, or something like that” (male resident, 83 years old). One participant also expressed an interest in more active VR experiences, much like scuba diving, suggesting “I wanna be in a fighter jet, I wanna fly, I wanna skydive, like you know, hopefully that will be possible in the future” (female resident, 80 years old). Finally, one participant stated “Well I’ll tell you what I’d like to say, and I think [naming of two other participants] both agree to this, that when this becomes more perfected […] Because I know you’re gonna use this to get the apps going much better […] so when all that is perfected, I would love to come back and do that again (female resident, 86 years old).

## Discussion

With emergent innovation and engineering technology, VR may provide an immersive tourism environment that allows users to experience a three-dimensional representation of a computer simulated environment. A significant advantage of VR tourism is that it is not constrained by physical, financial, or geographic boundaries that may impede older adults from engaging in lifelong tourism. Although preliminary in nature, findings from this study suggest that immersive VR tourism may provide an opportunity for continued learning, social engagement, and increased quality of life among older adults living in residential care settings.

In examining the immediate benefits of immersive VR tourism, a significant increase in momentary relaxation (decline in anxiousness) and a trending increase in momentary happiness was observed. The lack of statistical significance for momentary happiness may be due to happiness associated with anticipation of the VR session, which inevitably would create a ceiling effect. Qualitative reports suggested that the VR sessions increased happiness, describing a sense of feeling uplifted or “on a high” from the VR session. Although these findings are in contrast with [28], who reported null associations between VR exposure and change in observed affect, the difference in methodology (observation vs. self-report) make it difficult to draw clear comparisons.

The finding of minimal cybersickness in response to immersive VR content among older adults is especially noteworthy. Existing research has not explicitly investigated older adults’ tolerance of immersive VR in terms of cybersickness) [28, 33, 34]. VR tourism content between six to 10 minutes in length was chosen as a conservative approach to test tolerability to the immersive VR environment; which is slightly longer than exposure time in previous research with residential care participants [28]. Still, within this relatively short duration, cybersickness was not experienced. In fact, results indicated that immersive VR tourism decreased self-reported fatigue on the SSQ scale, which was further voiced qualitatively, with immersive VR having an energizing effect.

Following the 6-week VR tourism program, participants reported a significant increase in social engagement and quality of life. Although analysis failed to show a significant decline in self-reported depressive symptoms, it should be noted that the average score on the GDS was low, with a majority of the sample not endorsing clinically meaningful depressive symptoms, limiting the ability to detect changes over the course of the program. Qualitative findings supported the increase in social engagement, with some participants noting that VR tourism provided them with a conversation piece to discuss with family and friends and one participant shared that engaging in the VR program contributed to engagement in other activities that felt good. Enhanced quality of life was supported by qualitative reports of having something to look forward to, reports of learning and discovery, and reports of breaking the monotony of daily living in residential care.

Qualitative interviews provided some insight into the process by which VR tourism may enhance wellbeing. Indeed, qualitative reports alluded to three of the five core psychological mechanisms of wellbeing described by Newman et al. [10]: detachment-recovery, meaning, and affiliation. Detachment-recovery was expressed through shared stories of removing oneself from the monotonous nature of living in residential care. Detachment from the residential care environment provided a sense of escapism, which is a notable realm within the tourism experience [35] and is a unique motivation for travel among older adults [11]. Detachment-recovery supports relaxation and life satisfaction [36], which aligns with the study findings of enhanced momentary relaxation and increase quality of life. Some participants were captivated by the immersive experience and one participant expressed that VR tourism distracted them from their physical pain.

Meaningful leisure, or leisure that fosters the cultivation of something important or valuable in life [10], was expressed by participants who shared that the VR tourism program provided an opportunity to explore new horizons or to reminisce about past tourism experiences. Participants shared the joy of visiting places that they had never seen, to learn about different cultures, and to explore places that they otherwise would never be able to visit. Participants who had previously travelled to the VR destinations enjoyed reminiscing about their previous travels, which provided comfort, and reported that the VR experience allowed for an added dimension to their previous experience. Participants expressed the anticipation of travelling somewhere new each week. Of note, this sense of anticipation and “looking forward to” may have dampened study findings pertaining to momentary changes in happiness.

Finally, affiliation was alluded to in the qualitative interview. The VR tourism program provided participants with a shared experience of new travels, which not only elicited discussion between participants outside of the scheduled program, but further facilitated sharing of VR tourism content with friends and family. The ability to maintain social engagement within collective dwellings is paramount as research shows that level of social engagement predicts subjective and objective health status, mortality, and life satisfaction in older adults with disease and disability [37]. Consequently, immersive VR may provide a viable resource to promote affiliation among older adults living in collective dwellings. Together, preliminary findings suggest that VR tourism may provide a source of meaningful leisure which may enhance the wellbeing of older adults living in residential care.

To support future development of VR tourism, participants were asked to provide their critical feedback on VR tourism for older adults in residential care. Participants were enthusiastic about further development and improvement on the VR content and the immersive VR apparatus. Notable points of development included the quality and clarity of the videos, the importance of narration to facilitate presence and learning, the importance of culturally appropriate music that is aligned with the travel destination, and greater scope of filmed content. Although not a prominent point of discussion, the head-mounted display used in this study (Samsung Gear VR headset) caused slight discomfort for some of the participants who wore glasses. Of note, one participant withdrew from the study following the first exposure session due to discomfort associated with the VR headset. As such VR developers are encouraged to consider older adults in the design of their products.

The current findings are novel and provide a stepping-stone for future research on the benefits of immersive VR tourism for older adults in residential care. Given the preliminary nature of this study, the current findings must be interpreted in light of study limitations. First, the length of each video was relatively short, between six to 10 minutes in length. This length was considered too short by the participants, which may have limited the ability for participants to fully engage with the tourism content. Further, the 360-degree stock footage used in the present study consisted of existing files that were available for the study but were not created in a standardized way. Many participants noted the inconsistent quality of VR content, with some destinations providing a more full, immersive experience than others. Unsatisfactory content may have led to less involvement with the virtual environment. Of note, one participant dropped out of the study within the first week due to disappointment with the tourism content. To address this limitation, researchers are encouraged to partner with industry and employ participant engaged strategies in the development of VR tourism content for older adults.

A second limitation of this study was the small sample size, which may have resulted in Type II error and prevented the inclusion of potential covariates, including age, gender, visual and auditory impairments. Furthermore, multiple exploratory comparisons without statistical correction may have inflated the familywise Type I error rate. A third study limitation is that participants self-selected to participate in this study, with more males than females enrolled in the study. As such, results may not generalize to older adults who are less likely to engage in residential recreation activities. Finally, lack of a control group precludes causal inferences. Although qualitative data directly support quantitative changes in social engagement and enjoyment of immersive VR tourism, there may be other explanations for the quantitative findings, including natural fluctuations over time or engagement in a research project. Future randomized trials are necessary to determine whether benefits associated with VR tourism are greater than standard programming.

## Conclusions

These findings are novel as they suggest that immersive VR may provide the opportunity for older adults to continue to travel, albeit virtually, while still experiencing the enjoyment and satisfaction that a physical trip may afford. The findings from this study support the notion that meaningful leisure is an area deserving of increased empirical attention as it pertains to interventions for older adults living in collective dwellings. The rapid rate at which technology is advancing alongside the aging population will mean ample opportunity to incorporate technology into caring for older adults in the future.
